# Supporting the mental health of undergraduate women in chemistry

**DOI:** 10.1038/s42004-025-01806-6

**Published:** 2025-12-26

**Authors:** Isobel Grint, Loukia Taxitari, Marilena Mousoulidou, Andri Christodoulou, Rafaela Vasiliadou

**Affiliations:** 1 The Open University, School of Life, Health and Chemical Sciences, Walton Hall, Karen Hills, Milton Keynes, UK; 2 Neapolis University Pafos, Department of Psychology, Pafos, Cyprus

**Keywords:** Analytical chemistry, Electrochemistry

## Abstract

Mental health should be a priority for all and especially for underrepresented groups, including the women in chemistry. Here, the authors discuss and propose supportive mechanisms within academia for the mental health of undergraduate women in chemistry.

Women have made significant contributions to the demanding and innovative field of chemistry. Notable figures include Marie Curie (Noble Prize), Kamala Solonie, Rosalind Franklin, Irène Joliot-Curie (Noble Prize), Chengye Yuan, Uma Chondry and Helen M. Free. Their contributions shaped the landscape of today’s chemistry, providing valuable role models and sources of inspiration for thousands of women around the globe. However, despite the impactful presence of women, they continue to face underrepresentation in many areas of the field. According to statistics, only 14.13 of chemistry professors (full) in the UK are women^1^, highlighting a stark contrast compared to their male counterparts. As a community, it is vital to support the career progression of women starting from the undergraduate level and to build strong foundations that enable more women to attain senior positions. By supporting and empowering undergraduate women in chemistry, we pave the way for a more innovative future, attract talented women in leadership positions and increase diversity within the field, contributing significantly to economic growth. Improving the representation of women in research and leadership positions can break the stereotypes around the capabilities of women in STEM. At a personal level, women can benefit in career advancement through equal opportunities and policies. We propose that a tailored support focusing exclusively on undergraduate women can build the foundations of their successful future careers. Retaining and increasing the representation of women in chemistry requires an in-depth understanding of multiple barriers, as well as their intersectional identities, rather than focusing on a single contribution. Stereotypes, care responsibilities, financial support, ethnicity and race are a few examples of the barriers that have been investigated in recent years, in an attempt to increase participation within the broader context of female students in STEM and chemistry^2^. However, mental health and well-being must also be priority areas and a main focus in higher education if we truly wish to bring a new era of gender equity within the chemistry community.

## Mental health of women undergraduates in chemistry

Poor mental health is a common reason for students wanting to drop out of university^3^. Studies, which we note have focused on cisgender women, suggested that female students and young women are more likely to be diagnosed with a mental health condition^4–10^. In the United Kingdom, the mental health of students has become a growing concern^11^. Investigations before^3^ and during^6^ the COVID-19 pandemic have shown a persistent issue related to poor mental health, with female students being again the most heavily affected group population. A large-scale investigation (n = 1167) at a university in Northern England has shown that more than half of students (undergraduate and postgraduate) scored above the clinical cut off for depression and anxiety. Higher depressive symptoms were found in female students, as compared to male students. According to the authors, the reasoning behind the high levels of depression and anxiety might be due to the transition period that occurred during the first lockdown^12^. However, Allen et al.^13^ suggested that the increased levels of depression and anxiety persisted even after the first lockdown.

The Royal Society of Chemistry has reported the need for more data related to mental health within the chemistry community^14^, even before the onset of the pandemic. These strong indications suggest an urgent need for developing supportive mechanisms in higher education in order to improve the mental health of undergraduate women in chemistry. However, women represent a highly diverse group with intersectional identities^2^, making a one-size-fits-all approach often ineffective. While the current piece focuses on support mechanisms for undergraduate women, it is important to note that the applicability and impact of these recommendations can vary significantly based on factors such as race, class, disability, gender identity, and other sources of marginalization.

## Mental health support mechanisms for undergraduate women in chemistry

We propose that developing supportive mechanisms in higher education should enable undergraduate women to achieve their maximum potential and enjoy a balanced life. Herein, we discuss the potential benefits of (1) Networks for women, (2) Ongoing Research, (3) Awareness and training, (4) Campaigns.

### Networks for women

We feel that developing a friendly and supportive network is essential for the well-being of the undergraduate women. Chemistry departments should invest on the development of networks to promote well-being, socialization, and inspiration. The committee should be composed primarily of experienced women academics; however, the frequent participation of student representatives and professionals should be encouraged to address challenges that might arise. Main duties can involve the organisation of various social or sporting events, inspirational talks, and educational trips, in an attempt to reduce feelings of isolation and anxiety. Additionally, mentoring programmes can be offered within the network by matching students with women mentors for advice and guide. Furthermore, the network can collaborate with international and national networks to share experiences, challenges, and ideas. Engaging students to participate is vital for the success of the network, and measures should be taken to ensure active involvement. Thereby, universities could follow a strategy by posting frequently on social media (Facebook, Instagram, and Platform X, LinkedIn, and Intranet), updating relevant information on departmental boards, and mentioning the initiative during welcome talks at the beginning of each year. Networks can have a positive impact on the mental health and well-being of women students through mentoring, socialization, and continuous engagement in various activities, thereby creating a supportive and friendly environment. Furthermore, undergraduate women can receive emotional support by sharing their challenges and experiences, as well as socializing with peers, which helps reduce feelings of isolation.

### Ongoing research

Social and psychological sciences have already addressed structural barriers within the broader context of women in STEM. For example, investigations suggest that isolation and loneliness experienced by women in STEM workplaces hinder their long-term career progression due to the lack of a critical mass of women and unwelcoming environments^15,16^. Motherhood presents another common barrier, as employers often expect women to dedicate more time to family responsibilities compared to men^17,18^). Additionally, social expectations pressure women to reduce their working hours after childbirth^17,18^. Lack of mentorship and prevailing stereotypes are also significant structural barriers affecting both career advancement and academic success^16,19,20^. Furthermore, systemic barriers in research, including the funding progress contribute to their lower representation in more senior academic positions^2^. The effect is more prominent on women with intersecting marginalized identities such as race, ethnicity, and socioeconomic status. Women receive fewer and lower research grants compared to men, as described by the track-record emphasis bias, known as Mathew effect. In the UK, black women are heavily loaded with teaching, administrative, and organisational responsibilities, impacting their time for research bids in an already biased environment. As a consequence, women become more vulnerable on commitments outside research and thereby causing a significant decrease in the number of awarded research grants and in career progression. These important issues, barriers, and discriminations that have been described are affecting directly and indirectly the mental health of the broader context of women in STEM. Thus, effective solutions are required, starting from academia and especially at undergraduate level, which serves as the foundational stage for career development^16,20^. In the field of chemistry, a more targeted and interdisciplinary research is required, considering the persistent issue of poor mental health. The collaboration of psychologists and chemists is essential to identify impacts and improve the current situation through rigorous research, including surveys and interviews. Systemic issues such as tight schedules^21^, academic pressure for high grades, increased workload^21^, and demands of a constant work-life balance^22^ are some of the most common stressors that need further exploration in the upcoming years at the interface of chemistry and psychology.

Researchers interested in contributing to this interdisciplinary area also face systemic issues. We believe that grant schemes designed exclusively for the inclusion, diversity, equity, justice, and empowerment should be offered, in which group leaders and PhD students can conduct high-quality research on relevant topics. Grants and funding opportunities can advance knowledge by contributing to the development of new strategies to improve and maintain mental health. Most importantly, they highlight the significance of prioritizing mental health and well-being within the academic community. Also, journal dedicated to this topic, as well as conferences, could bring a supportive environment for researchers and future work. Unfortunately, currently those sources are very limited, making it extremely challenging for the interested researchers. Increasing both the availability and visibility of these resources can support mental health education and awareness. In terms of research and funding process, minority groups, including women with intersectional identities, should be prioritized from the undergraduate levels. Creating inclusive research environments can be achieved through the creation of summer undergraduate research internships for minority groups.

## Awareness and training

Faculty and staff must receive adequate training on how to identify signs of distress in students. Academics, tutors, and teaching assistants are usually the first to observe certain behaviours due to their daily exposure to students through lectures, labs, and meetings. Identifying the signs of distress is vital since academics can recommend that students seek professional help using mental counselling services within universities. Proactive mental health training is essential since it can prevent the onset of mental issues and improve the well-being of students. The training must be mandatory and subject to promotion, so as to equip all educators with this valuable skill, rather than being an optional requirement. Students’ mental health should be observed in all modules for a complete picture. A hybrid mode of training would enable flexibility and accommodate the needs for each trainee. A dedicated team of experts should always be in close communication with the academics and related staff, especially in cases of a suspected mental health issue, so as to provide the necessary support and future steps for professional help. Academic staff should also be train on the development of inclusive environment through mandatory training involving workshops and coursework.

## Campaigns

Campaigns are ideal tools for promoting awareness of poor mental health and play a crucial role in reducing feelings of embarrassment and shyness among the student population. Universities should organize mental health campaigns to educate students, reduce stigma, and normalize open discussions around mental health topics. Such initiatives can encourage students to seek professional help, manage their mental well-being effectively, and adopt a balanced lifestyle. Moreover, campaigns can provide valuable platforms to promote inclusivity, gender equality, and foster mentorship and leadership development, particularly among women in chemistry. A notable example is the IUPAC Storytelling Campaign, which was an excellent initiative as part of the IUPAC Global Women’s Breakfast 2025^23^. This campaign successfully addressed these critical aspects by educating participants and breaking down stereotypes within the scientific community. Universities can adapt similar campaigns by incorporating mental health awareness and student well-being as integral components. Furthermore, collaboration among chemistry departments to organize larger, joint campaign events can significantly increase awareness and create a dynamic network. This approach not only encourages greater student participation as part of a larger community but also helps reduce feelings of shyness and isolation.

## Conclusion

Women in chemistry should be a highly diverse and dynamic group, welcoming all undergraduates who feel that they belong here, regardless of their intersectional identities. The only requirement is their passion and love for chemistry. A friendly, supportive, and comfortable environment without restrictions or boundaries. Studying for a chemistry degree is already challenging and requires effort and hard work; let’s make this journey equal to all. The chemistry community cannot afford to lose any more talented chemists. As a consequence, mental health and well-being should be prioritized within the chemistry community. Increased levels of anxiety and depression suggest an urgent need to develop targeted supportive mechanisms within the coming years. We hope that this piece provides food for thought and a useful starting point for colleagues in university chemistry departments to implement mental health support mechanisms for women and all minority groups. Future work requires a deep investigation on the mental health of all the underrepresented groups, considering as well as their intersectional identities, towards the creation of a truly inclusive environment.
